# Our California Letter

**Published:** 1869-02-15

**Authors:** 


### *Our California Letter.*

San Francisco, Cal., Jan. 13.

Dear Editor:—I promised you some leaves from my note book on the Pacific coast, and after a sojourn of only a few weeks find myself charged with material for at least a dozen pseudo-professional letters.

Among the California pioneers one soon comes to feel that to reach San Francisco by one of Pullman's sleeping cars will be an inglorious mode of entering the country. As the German said to his newly-arrived friend—"Coom'd you de isthmus mit?" "Nein." "Oh! den you coom de blains across?" "Nein." "Vy! den you cooms de Horn around?" "*Nein.*" "Vot a dricks! *Den you no cooms at all!*" Transit by cars, sleeping one half the time, eating and reading newspapers a good part of the other half, always on the wrong side when anything is to be seen; however desirable in a commercial point of view, this will be loss of many of the chief advantages of travel. Give us rather the prairie schooner, or the long sea voyage, from which the Golden Gate opens suddenly into a land of promise and delight.

In that part of the earth created and furnished expressly with reference to our noble profession, *Central America*, I lingered only long enough to resolve to visit it again, with full leisure to observe the diseases born of the over luxuriance of nature; and the vast supplies of remedies, as yet only half developed, with which it is stored. No doubt a more faithful study of indigenous practice would give many valuable hints for the successful treatment of those destructive miasmatic fevers which have so long baffled the enterprise of connecting the oceans, and but for which thriving cities would take the place which squalid Aspinwall and sleepy Panama now occupy on the map of the world.

The popular idea that either of these localities are eminently breeding-places of the Panama fever is, I think, an erroneous one. The fact is, the whole country, from shore to shore, least of all where exposed to the ocean breeze, is reeking with miasm, and a rapid journey of less than four hours, in clean, well-ventilated cars, in the day time, and at the most favorable season of the year, will often be followed by an attack. Several of our ship's company who did not indulge in tropical fruits, or in sight-seeing at either terminus, suffered, some of them after their arrival at San Francisco. In the greater number of cases there has been imprudence in diet, and exposure, but the confinement of the ship, and change from ordinary habits of living are in themselves sufficient predisposing causes.

The natives of the Isthmus, especially the American of unmixed blood, have little to fear from anything but exotic diseases. When the fever seizes them, they take a heroic dose of the Jatropa or Physic Nut, and follow this with liberal potations of "Copatchi," an infusion of bark in Jamaica rum, which produces exactly the effect of quinine. This excellent medicine is offered for sale upon the cars, but I am sorry to say that the majority seem to prefer it without the bark. An old medical friend who has practiced, and been practiced upon, for some years in Guatemala, informs me that he repeatedly conquered attacks of fever by violent exercise, literally running for his life, and following up the profuse perspiration thus induced with the aforesaid bitter infusion. He became thoroughly acclimated not only to fever, but to the scorpion, tarantula and "Tommy Goff," not to mention certain winged abominations which drive even naturalists out of the country.

Perhaps it is well that the sinuous strip of tropic land in our hemisphere should remain undisturbed, to show what nature can do in the vegetable line.

Man seems a pigmy in the presence of these mighty forms. The stately colonades of palm, the lofty ceibas and mahogany trees with their hanging gardens of parasites, and the infinitely varied luxuriance of undergrowth, defy the best attempts of pen or pencil. They must be seen to be realized, and with sight comes the conviction that the outward senses are not strong enough, nor the spiritual senses fine enough to apprehend their beauty. The marvel of the tropics is the "silk cotton tree," which often grows to the height of ninety feet. Its giant stems are used by the natives for canoes, and as it branches near the top, and throws out long and massive limbs, it is, in the floweriug season an enormous flat bouquet, an ærial field of fine carnation relieved by the deepest green.

The banana, which in a given extent of ground affords forty-four times more nutritive material than the potato, and one hundred and thirty-three times more than wheat, seems the most appropriate symbol of lands so rich in food, medicine, precious woods and gums. Were I a believer in "similia similibus" I would not trust to the efficacy of counter effects as a protection against the dangers of these *drug* forests, any quarter-section of which will furnish aloes, copaiba, cassia, camphor, dragon's blood, ipecac, jesuit's bark, and musk enough to freight a ship.

Precious gifts have they also of spices (pimento gordo and cassia), of frankincense and myrrh; one cannot but wonder that some enterprising Yankee has not established a manufactory of pure extracts in Central America.

We arrived in San Francisco too late to enjoy the earthquake, or to verify without some painstaking the reports we had heard of its ravages—the Franciscans like the Chicagonese think more of the good name of their city than of their personal convenience or safety.

A peculiar physical sensation, not unlike that of fear is admitted to have prevailed during the month of October. It affected the appetite and circulation, and even the locomotive organs; furnished no end of "items", and gave way in the public mind to the "small-pox", which could not and would not be treated as a freak of electricity. Many minds that gravely doubted whether the influence which sent the chimneys over into the streets, and church-steeples crashing down upon the pews, was the result of any action upon or below the earth's crust, were impressed by a fear that the advent of a new disease imported direct from China would prove dangerous if not watched over by the proper authorities. Yellow flags signified, in a very timid and deprecatory manner, the presence of infection, while the plague spread until the infection of nearly every precinct made the most stringent measures necessary. There is no disguising the facts that the mortality from small-pox during the three last months in San Francisco has been appalling—and that a larger number than is usual of fatal cases have been vaccinated, and not unfrequently have had the disease before. It has not raged particularly among the Chinese—they have not suffered as much as the poorer classes of other nationalities. It has been claimed by the papers that the Chinese doctors have been far more successful than others in their treatment, which I can believe, knowing one of their fundamental principles is that "disease proceeds from imperfect irrigation of the body." Why any body should be imperfectly irrigated in this city is not so clear, the most abundant facilities being offered. It is rather difficult to find out exactly what the Chinese mode of treatment is, bnt as the epidemic is abating, and Doctor Liang Wantai Scincang, (which being interpreted signifies "terrace of letters,") speaks the English tongue. I hope to understand the rationale of it. If what Remusat says of this singular people is true—"instead of studying the organization of bodies they undertake to determine by reasoning how they ought to be"—one would expect a kind of metaphysical treatment consisting in "wholesome letting alone," and in this possibly lies the secret of success.

Like other highly civilized nations the Chinese have their opposing schools of medicine, and these are well represented among the Anglo-Chinese. Drs. Chan Lin Phory, Chong Po Chi, and Hab Ling, possess the credentials of the Hanlen Quen or Imperial Academy, have a large practice, and may yet appear as delegates to the National Medical Association.

The smell of carbolic acid reminds us often of old Philip Frenau's verse—"Safe in the atmosphere of scents."* Much of the article offered for sale has nothing genuine except the smell. The accomplished President of the Catholic college at Santa Clara, as erudite in chemistry as in theology, offered to us the genuine "Phenic," pure as the best French processes could make it. With him and his army of bright, intelligent boys we have spent our pleasantest hours in California. Nowhere else have we seen science so entirely pervading the system of education. Of the honorable fraternity of teachers and doctors in the Golden State we shall speak in another letter.

E. S. C.

* "On prancing steed, with sponge at nose,
From town behold Sangrado fly.
Camphor and tar, where'er he goes,
The infected shafts of death defy—
Safe in an atmosphere of scents,
He leaves us to our own defence."

PHILIP FRENAU, 1793.

